# Editorial: Non-canonical nucleic acid structures, functions and their applications for understanding human genetic diseases

**DOI:** 10.3389/fgene.2023.1188978

**Published:** 2023-04-05

**Authors:** Roshan Satange, Peng Jin, Ming-Hon Hou, Ambadas B. Rode

**Affiliations:** ^1^ Institute of Genomics and Bioinformatics, National Chung Hsing University, Taichung, Taiwan; ^2^ Department of Human Genetics, Emory University School of Medicine, Atlanta, GA, United States; ^3^ Department of Life Sciences, National Chung Hsing University, Taichung, Taiwan; ^4^ Regional Centre for Biotechnology, NCR Biotech Science Cluster, Faridabad, India

**Keywords:** non-canonical nucleic acids, genetic diseases, cancers, neurological diseases, functional nucleic acids

Nucleic acids, deoxyribonucleic acid (DNA) and ribonucleic acid (RNA), are macromolecules that are essential for all known forms of life. Besides storing genetic information, nucleic acids regulate key biological processes inside the cell, including genes expression, DNA replication, recombination and repair. They are also involved in diseases such as cancer and neurological disorders therefore represent potential drug targets. The DNA and RNA execute their regulatory functions by adopting diverse secondary and tertiary conformations which are essential for specific molecular recognition of their cognate targets. Nucleic acids adopt diverse conformations through several types of hydrogen bonding patterns. The canonical double-stranded structure consists of two antiparallel strands intertwined by Watson-Crick A-T and G-C base pairs. On the other hand, the non-canonical structures include G-quadruplexes, i-motifs, triplexes and cruciform hairpins, etc. are formed by alternative base pairings such as Hoogsteen and Wobble base pairs. Given their crucial biological functions, non-canonical structures are of great interests in genetics, molecular biology and drug discovery.

The ability of nucleic acids to adopt different conformations depends on the sequence. The abnormal expansion of certain nucleic acid sequences has been linked to numerous neurological diseases. For example, a hexanucleotide repeat expansion mutation in chromosome 9 open reading frame 72 (*C9orf72*) gene causes neurodegenerative diseases, amyotrophic lateral sclerosis (ALS) and frontotemporal dementia (FTD), through the formation of non-canonical nucleic acid structures in the genome. Recent technological advances have uncovered the additional functional roles of non-canonical nucleic acids in the pathobiology of the diseases. Using high-throughput genomic, structural and computational methods, non-canonical nucleic acid structures have emerged as promising tools for various applications such as DNA nanotechnology, gene therapy and drug discovery. For this Research Topic, we were looking for research studies describing new insights into the structures and functions of non-canonical nucleic acids, their understanding in regulation of biological processes, and relation to different human genetic diseases including cancer and neurological disorders.

This theme could therefore include a range of topics dealing with structural and functional studies of disease-relevant non-canonical DNA/RNA structures and their biological association in cellular processes such as epigenetic and transcriptional regulation. The final Research Topic has published five articles covering a wide range of research areas on non-canonical nucleic acids. For example, Bansal et al. have extensively reviewed the polymorphic features of non-canonical DNAs, including single-, double-, triple- and four-stranded structures, and their association with genetic instabilities and major human diseases. Although much of the literature is available on multi-stranded non-canonical structures such as G-quadruplexes, the secondary non-canonical structures formed by double-stranded DNAs are often overlooked. Bansal et al. therefore also focused on the polymorphic nature of left-handed Z-DNA structures and their interactions with Z-DNA-binding proteins, and explained how the formation of Z-DNA structures within the genome is related to genetic instabilities, chromosome breaks and translocation-related human diseases. In addition, the authors highlighted current and future prospects for the use of non-canonical DNAs such as aptamers in disease therapy. In another article of this Research Topic, Yousuf et al. provided a detailed overview of the role of non-canonical DNA and RNA structures in the context of fragile X-related neurological disorders, Fragile X-associated tremor/ataxia syndrome (FXTAS) and Fragile X syndrome (FXS). Although these disorders have different clinical manifestations as well as different molecular pathogenesis mechanisms, it is known that these disorders are caused by aberrant expansion of CCG repeat sequences in the fragile X messenger ribonucleoprotein 1 (FMR1) gene through the formation of non-canonical secondary structures such as hairpins and loops. The authors have conscientiously provided the reader with a detailed review from the available literature of possible molecular mechanisms of repeat instabilities and the role of secondary structure formation responsible for the FXTAS and FXS. In addition, Yousuf et al. outlined how new technologies such as whole-genome analysis of short tandem repeats could identify genes for these diseases and the potential for targeting the disease-related genes or their respective transcripts for therapeutic purpose. They concluded that a more detailed understanding of secondary structure formation and its downstream effects would be helpful to describe the mechanisms behind the pathobiology of the fragile X related diseases and to aid in the development of therapeutic strategies against these disorders.

As mentioned above, G-quadruplexes are one of the best-studied multistranded non-canonical structures found in the genome and are involved in the regulation of processes such as transcription, translation, replication, etc. Nevertheless, the exact role and functions of G-quadruplexes in transcriptional regulation as well as epigenetic modifications have remained unclear, especially their interactions with transcription factors. To address this gap, Fang et al. performed an integrative analysis of G4 multiomics data derived from high-throughput ChiP sequencing and *in silico* analyses. In this study, the authors found a strong correlation between gene expressions and the G-quadruplex-forming DNA strand as well as the location of G-quadruplex forming sequences in those genes. This study thus makes an important contribution to the existing knowledge on G-quadruplexes and their genetic linkage. It has also been found that the epigenetic modifications such as methylation can influence gene expression by affecting transcription factors binding. In a study by Zhu et al., the authors proposed a correlation between methylation and methylation-based gene prediction signatures in cancer, using next-generation sequencing data and DNA methylation profile analyses. The authors concluded that the signatures predicted in this study could be useful as potential prognostic biomarkers as well as therapeutic targets in certain cancers. In addition to these DNA structures, non-canonical RNA structures are also crucial for cellular processes such as RNA processing and localization together with protein complexes. For example, the U5 snRNA is a component of the ribonucleoprotein complex that processes the splicing of premature mRNAs. In a comprehensive review article by Wood et al., the authors defined the role of various ribonucleoproteins associated with craniofacial disorders as well as cancer and their disease mechanisms.

In conclusion, the molecular understanding of non-canonical nucleic acids has opened new avenues of investigation into their roles in biological processes and disease pathogenesis. Studies published as part of this Research Topic have revealed a complex world of alternative nucleic acid structures and functions that differ from the classical double helix structure.

